# CX3CR1 and CCR2: dynamic myeloid cell states across inflammatory diseases with implications for oral health and disease

**DOI:** 10.3389/fimmu.2026.1873474

**Published:** 2026-06-17

**Authors:** Yun-Ji Lim, Tae Sung Kim

**Affiliations:** 1 Department of Biochemistry, School of Medicine, Pusan National University, Yangsan, Republic of Korea; 2 Research Institute for Convergence of Biomedical Science and Technology, Pusan National University Yangsan Hospital, Yangsan, Republic of Korea; 3 Department of Oral Microbiology, School of Dentistry, Dental and Life Science Institute, Pusan National University, Yangsan, Republic of Korea; 4 Education and Research Team for Life Science on Dentistry, Pusan National University, Yangsan, Republic of Korea

**Keywords:** CCR2, CX3CR1, mucosal immunity, myeloid cell states, periodontitis

## Abstract

Chemokine receptor-mediated immune cell trafficking is a fundamental determinant of inflammatory disease outcomes. Among chemokine receptors, CCR2 and CX3CR1 represent two functionally distinct but interconnected axes that regulate inflammatory monocyte recruitment and tissue macrophage retention, respectively. However, emerging evidence suggests that these receptors function as dynamic regulators of context-dependent myeloid cell states rather than fixed markers of discrete immune cell subsets. A central focus of this review is the recruitment-to-residency axis, which describes the functional transition of myeloid cells from inflammatory recruitment to tissue adaptation during chronic inflammation. In acute inflammation, CCR2-dependent recruitment is often essential for host defense and tissue repair; however, sustained CCR2 signaling promotes chronic inflammation and tissue damage. Conversely, CX3CR1 signaling maintains homeostasis under steady-state conditions, but can contribute to disease chronicity by retaining pathogenic macrophage populations within tissues. In this review, we summarize the cellular sources and regulatory mechanisms of CX3CR1 and CCR2, discuss their context-dependent roles across neurological, cardiovascular, mucosal, metabolic, fibrotic, and infectious diseases, and propose a unifying recruitment-to-residency axis. We further discuss periodontitis as a representative chronic inflammatory disease in which dysregulated CCR2-CX3CR1 dynamics may contribute to persistent inflammatory macrophage accumulation and osteoclastogenic tissue destruction. Understanding the dynamic interplay between CX3CR1 and CCR2 provides a conceptual framework for precision immunomodulatory strategies targeting the transition points of chronic inflammatory diseases, including those affecting oral health.

## 1 Introduction

The outcome of inflammatory diseases is not solely determined by the magnitude of immune cell recruitment, but by the functional states, persistence, and tissue adaptation of infiltrating cells (1, 2). While classical models have emphasized the expansion of discrete immune cell subsets, emerging evidence indicates that myeloid cells instead operate along dynamic and context-dependent trajectories shaped by local environmental cues (3, 4). Defining these state transitions is therefore essential for understanding how inflammation resolves or progresses toward chronic pathology. This review moves beyond traditional lineage-based classifications to provide a novel integrative perspective on myeloid plasticity, focusing on the transition between recruitment and residency.

Chemokine receptors play a central role in orchestrating these processes by regulating leukocyte trafficking, retention, and differentiation across tissues (5–7). Among them, CCR2 and CX3CR1 have traditionally been viewed as prototypical markers of inflammatory monocytes and tissue-resident macrophages, respectively (8–11). However, we argue that this binary classification oversimplifies the functional complexity of myeloid responses. Recent studies in fate-mapping and single-cell technologies suggest that CCR2 and CX3CR1 delineate a dynamic continuum of myeloid cell states that evolve functionally as they transit through different tissue compartments during the course of inflammation (12–14). In this recruitment-to-residency axis, CCR2^+^ inflammatory monocytes progressively acquire CX3CR1 expression as they adapt to the tissue microenvironment, transitioning from pro-inflammatory effectors to tissue-resident or regulatory populations (15, 16).

A key objective and unique contribution of this review is to bridge the gap between general systemic immunology and oral pathobiology. We synthesize current knowledge on the regulatory mechanisms of the CCR2-CX3CR1 axis, and propose a unifying framework that defines these receptors as indicators of functional states rather than fixed subsets. Critically, we highlight periodontitis not merely as a localized infection, but as a prototypical mucosal inflammatory disease where the breakdown of the recruitment-to-residency axis drives pathological bone loss. By examining the synergy between CCR2-driven monocyte influx and CX3CR1-mediated persistence, we offer a new conceptual foundation for precision immunomodulatory strategies in oral and systemic health.

## 2 Cellular sources and myeloid states associated with CX3CR1 and CCR2

CX3CR1 and CCR2 are expressed across multiple immune cell types; however, their functional impact in inflammatory diseases is most prominently manifested within the myeloid compartment, particularly among monocytes and macrophages (9, 17, 18). Moving beyond the traditional view of these receptors as static markers of discrete cell lineages, this review defines them as indicators of dynamic and context-dependent myeloid cell states that evolve in response to tissue-derived signals and inflammatory cues (3, 19, 20).

**Figure 1:**
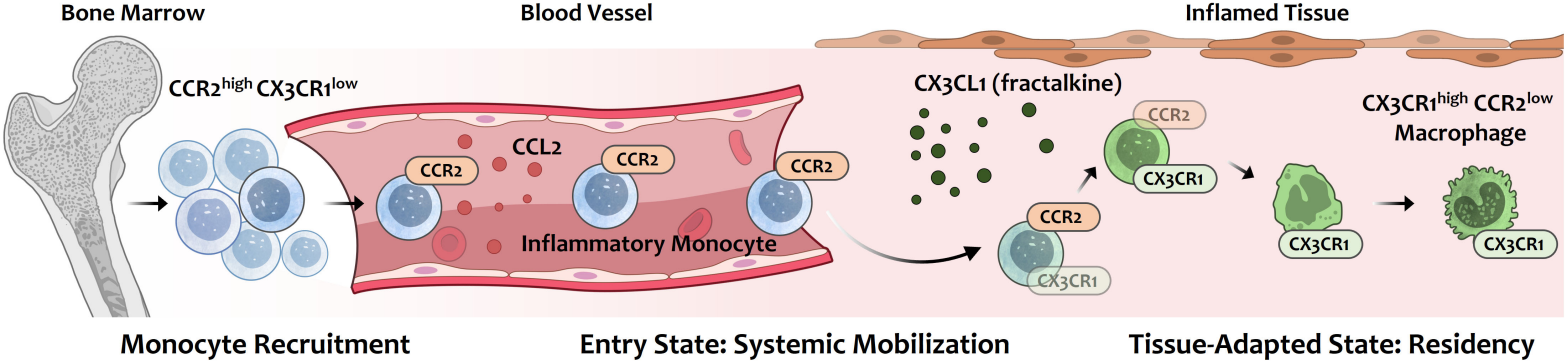
CCR2-CX3CR1 recruitment-to-residency axis in myeloid cell-state transitions. Schematic of the recruitment-to-residency axis, where CCR2^+^ inflammatory monocytes transition into CX3CR1^+^ tissue-adapted macrophages under microenvironmental cues, representing under physiological tissue adaptation conditions.

Within this framework, CCR2 and CX3CR1 delineate a functional continuum rather than mutually exclusive subsets (19, 21). CCR2 is predominantly associated with an ‘entry state’ characterizing recently recruited, inflammatory monocytes, whereas CX3CR1 marks a ‘tissue-adapted state’ exhibiting enhanced survival, retention, and functional specialization. A central theme of this discussion is the ‘recruitment-to-residency axis’, a model that captures the progressive shift of infiltrating cells from a migratory effector phase (CCR2^+^) to a stable, niche-adapted residency phase (CX3CR1^+^) (**Figure 1**).

In this section, we first outline the major immune cell populations expressing CX3CR1 and CCR2 and then discuss how their differential expression captures distinct yet interconnected functional states within the myeloid lineage. This integrative approach provides a unique foundation for understanding how dysregulated state transitions, rather than just cell numbers, drive chronic inflammatory pathologies like periodontitis.

### 2.1 CX3CR1-expressing cells: tissue-adapted macrophage states in homeostasis

CX3CR1 is expressed across diverse immune cell populations, including subsets of monocytes, macrophages, dendritic cells, CD8^+^ T cells, and natural killer cells (11, 22). However, its most prominent and conserved function emerges within tissue-resident macrophages, where CX3CR1 serves as a definitive indicator of successful tissue adaptation, longevity, and niche-specific immune surveillance (8, 23). Rather than being a static marker, CX3CR1 expression captures a specific functional state within the myeloid continuum, marking the transition from a migratory cell to a stable tissue sentinel (9, 19).

Across organs, CX3CR1^+^ macrophages exhibit a shared functional signature characterized by niche-specific specialization and homeostatic regulation (24, 25). In the central nervous system, CX3CR1 is highly expressed by microglia, embryonically derived macrophages originating from yolk sac progenitors (26, 27). CX3CR1 signaling in these cells regulates neuronal survival, synaptic pruning, and neuroimmune communication, thereby maintaining neural circuit homeostasis (27–30). Similarly, in peripheral tissues such as the liver, gut, lung, kidney, and heart, CX3CR1^+^ macrophages act as local sentinels that integrate environmental signals with immune regulation (31, 32).

Despite differences in ontogeny and tissue localization, these CX3CR1^+^ populations converge on several core functions (24). In barrier tissues such as the gut and liver, CX3CR1^+^ macrophages sense and restrict microbial dissemination, coordinate adaptive immune responses including IgA production, and form a frontline defense against environmental exposure (33–38). In the lung and kidney, CX3CR1^+^ macrophages support immune surveillance, regulate inflammatory resolution, and maintain tissue integrity through epithelial cross-talk and immune network stabilization (15, 39–41). In the heart, CX3CR1^+^ macrophages contribute to myocardial homeostasis, clearance of dying cells, and orchestration of tissue repair following injury (42–44).

Collectively, CX3CR1-expressing macrophages constitute a conserved, organ-spanning surveillance system that integrates tissue-specific cues with immune regulation. Importantly, in the context of our recruitment-to-residency axis, CX3CR1 expression reflects not merely lineage identity but a specialized state of tissue adaptation. This concept uniquely positions CX3CR1^+^ macrophages as the critical endpoint of myeloid differentiation, where cells have successfully transitioned from systemic recruitment to long-term tissue residency (**Figure 1**).

### 2.2 CCR2-expressing cells: inflammatory monocyte mobilization and tissue recruitment

CCR2 is predominantly expressed by inflammatory or classical monocytes and serves as a central regulator of their mobilization, trafficking, and functional deployment during inflammatory responses (45, 46). In mice, CCR2 is highly expressed on Ly6C^high^ monocytes (10, 23), whereas in humans it is most strongly associated with CD14^high^ classical monocytes (47, 48). Rather than viewing these as a fixed subset, we define these CCR2^+^ monocytes as a dynamic entry state within the myeloid continuum, specialized for rapid recruitment from systemic circulation to sites of tissue injury, infection or stress.

At the level of hematopoietic output, CCR2 signaling is essential for monocyte egress from the bone marrow (45, 46). Through sensing gradients of its primary ligand, CCL2 (MCP-1), as well as related ligands such as CCL7 (MCP-3) and CCL12 (MCP-5 in mice), CCR2 drives the release of monocytes into the circulation and coordinates their subsequent recruitment into inflamed tissues (46, 49). This process is tightly regulated by inflammatory cues, including pathogen-associated molecular patterns (PAMPs), cytokines such as TNF and IL-1β, and tissue-derived chemokine production, which collectively establish chemotactic gradients that guide CCR2-dependent trafficking (50, 51). Beyond its role in chemotactic recruitment, CCR2 signaling activates downstream inflammatory pathways that regulate monocyte survival, adhesion, and effector activation (50, 52). Ligand binding to CCR2 induces G-protein-coupled signaling cascades involving Phosphoinositide 3-kinase (PI3K)/Protein kinase B (Akt) signaling, Mitogen-activated protein kinase (MAPK)/Extracellular signal-regulated kinase (ERK), and Nuclear factor kappa-light-chain-enhancer of activated B (NF-κB) pathways, promoting cytoskeletal rearrangement, chemotaxis, inflammatory cytokine production, and heightened responsiveness to local inflammatory cues (53–56). Sustained CCR2 activation may therefore amplify inflammatory recruitment while priming infiltrating monocytes for pathogenic differentiation within chronically inflamed tissues (46, 57). Notably, this ligand-driven ignition phase sets the stage for the subsequent functional evolution of the cell within the tissue microenvironment (19, 58).

Upon entry into peripheral tissues, CCR2^+^ monocytes adopt highly plastic functional programs that are shaped by the local microenvironment (19, 58). In early phases of inflammation, these cells differentiate into pro-inflammatory macrophages or monocyte-derived dendritic cells, producing cytokines, reactive oxygen species, and antimicrobial effectors that contribute to host defense and tissue remodeling (59, 60). However, sustained or dysregulated CCR2-dependent recruitment results in the accumulation of inflammatory monocytes and their progeny, amplifying tissue damage and promoting chronic inflammatory pathology (50).

**Figure 2:**
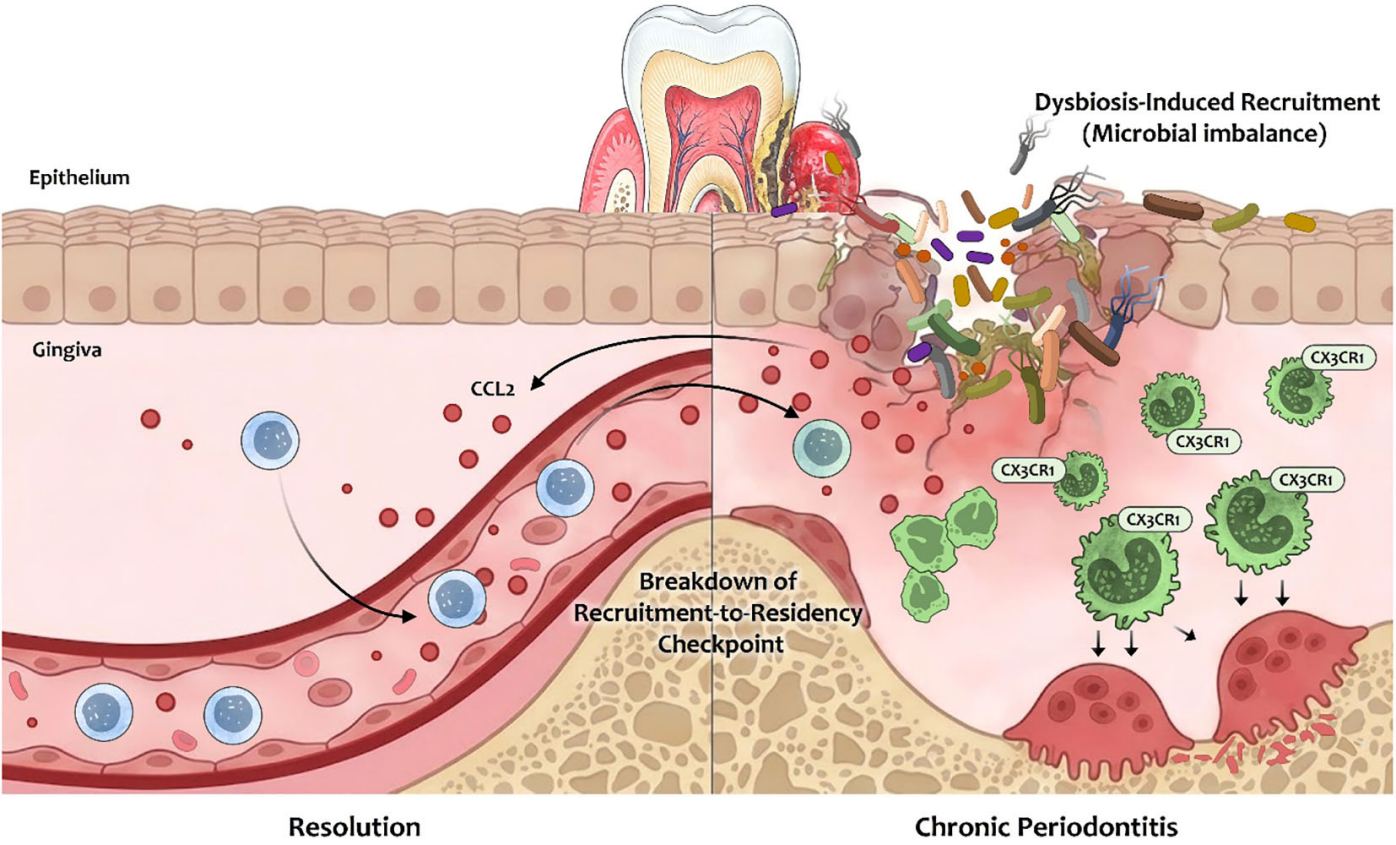
Pathological imbalance drives periodontitis. Integrated model of the CCR2-CX3CR1 axis showing ligand-driven recruitment-to-residency axis contrasting balanced inflammatory resolution under physiological conditions with pathological macrophage persistence, chronic inflammation, and alveolar bone loss.

The most critical and unique aspect of CCR2 biology in our model is that its expression is transient and serves as a functional checkpoint (45, 50). Recruited monocytes progressively downregulate CCR2 while acquiring alternative receptor programs, including increased expression of CX3CR1, as they undergo tissue adaptation and differentiation (9, 23). This transition reflects a fundamental shift from a migratory, inflammation-driven entry state toward a more resident and functionally specialized endpoint (**Figure 2**). Thus, CCR2^+^ monocytes represent the essential starting point of the recruitment-to-residency axis, linking systemic inflammatory signals to the establishment of long-term tissue niches, a process that becomes pathologically hijacked in diseases like periodontitis.

Collectively, CCR2 defines a dynamic and context-dependent myeloid state of inflammatory readiness and mobilization, redefining CCR2^+^ monocytes not merely as migratory effectors, but as the foundational entry state of the myeloid continuum and the essential precursors from which tissue-adapted macrophage populations emerge during the progression of inflammation.

## 3 CX3CR1 and CCR2 as markers of dynamic myeloid cell states

Accumulating evidence indicates that CX3CR1 and CCR2 should not be interpreted as markers of fixed myeloid lineages, but rather as indicators of dynamic and context-dependent cell states that evolve during inflammation. This shift from a lineage-based to a state-based framework has been driven by advances in fate-mapping, single-cell transcriptomics, and *in vivo* imaging, which collectively reveal that myeloid cells undergo continuous functional reprogramming in response to tissue-derived cues (**Figure 1**).

Recent single-cell and spatial transcriptomic studies in human periodontitis have further reinforced the concept of dynamic myeloid state transitions within inflamed periodontal tissues (61–63). These high-resolution analyses have identified previously unrecognized macrophage and monocyte subpopulations characterized by distinct inflammatory, osteoimmune, and immuno-metabolic signatures, while also revealing spatially organized inflammatory niches associated with chronic periodontal destruction (62, 63). Emerging multi-omics datasets further suggest that CCR2-CCL2 and CX3CL1-CX3CR1 signaling networks are integrated within broader stromal-immune communication circuits that regulate macrophage persistence, inflammatory amplification, and tissue remodeling in periodontitis (61, 63).

In many inflammatory settings, CCR2^+^ inflammatory monocytes are recruited from the circulation into tissues, where they encounter a complex microenvironment composed of cytokines, growth factors, microbial signals, and damage-associated molecular patterns (DAMPs) (50, 64). These signals initiate a progressive reprogramming process that reshapes transcriptional, metabolic, and functional profiles of infiltrating cells (65, 66). Crucially, this environmental encounter acts as a functional catalyst that drives the transition along the myeloid continuum (19).

Following tissue entry, recruited monocytes frequently downregulate CCR2 expression while concomitantly upregulating CX3CR1, marking a transition from a migratory, inflammation-driven state toward a tissue-adapted macrophage phenotype (9, 23). In addition to mediating cellular retention, CX3CR1 signaling regulates intracellular pathways associated with macrophage survival, tissue adaptation, and long-term residency (67, 68). Engagement of CX3CR1 by CX3CL1 activates PI3K-Akt, Src-family kinase, MAPK/ERK, NF-κB, and anti-apoptotic signaling pathways that enhance cellular survival, resistance to inflammatory stress, and stable adhesive interactions within tissue niches (67, 69, 70). These signaling programs collectively reinforce the stabilization and persistence of tissue-adapted macrophage states within chronically inflamed microenvironments. Rather than representing a binary switch, this process occurs along a dynamic continuum of intermediate states characterized by the progressive loss of migratory potential and the acquisition of tissue-specific functions, including enhanced tissue persistence, immune regulation, and niche-specific specialization (**Figure 2**).

However, emerging evidence suggests that this CCR2-to-CX3CR1 transition is not universally linear or uniform across all inflammatory settings (19, 20). Under certain pathological conditions, inflammatory monocytes may transiently co-express both CCR2 and CX3CR1, while some tissue-resident or tissue-adapted macrophage populations can re-express CCR2 in response to inflammatory reactivation (16, 71). In addition, early induction of CX3CR1 expression has been observed in activated inflammatory monocytes before complete loss of CCR2-associated migratory programs (10). These observations indicate that CCR2 and CX3CR1 should not be interpreted as mutually exclusive states, but rather as dynamically overlapping and context-dependent programs (19). The extent and directionality of these transitions likely vary according to tissue type, inflammatory duration, microbial exposure, stromal niche composition, and species-specific immune organization (65, 72). Therefore, our recruitment-to-residency model should be viewed as a conceptual framework that captures dominant trajectory patterns rather than a strictly irreversible or universally conserved linear sequence, with the precise trajectory being shaped by local microenvironmental conditions.

Mechanistically, this CCR2-to-CX3CR1 transition is actively regulated by local microenvironmental cues rather than occurring as a passive phenotypic conversion (25, 65). After tissue entry, recruited monocytes are exposed to inflammatory cytokines, growth factors, microbial products, hypoxic stress, and stromal niche-derived signals that collectively drive transcriptional and metabolic reprogramming. Factors such as M-CSF, IL-10, TGF-β, and tissue-derived survival signals promote macrophage differentiation and long-term adaptation (65, 73–75), while persistent inflammatory stimulation progressively suppresses CCR2-associated migratory programs and enhances CX3CR1 expression associated with tissue retention and survival (67, 76). This coordinated reprogramming ultimately shifts recruited monocytes from transient inflammatory recruitment programs toward stable tissue-adapted macrophage states characterized by long-term persistence and niche-specific specialization. At the molecular level, this process is accompanied by activation of lineage-defining transcriptional regulators, including Purine-rich box 1 (PU.1), MAF BZIP transcription factor B (MafB), Nuclear Receptor Subfamily 4 Group A Member 1 (NR4A1), and Krüppel-like factor 4 (KLF4) (19, 77–79), as well as epigenetic remodeling that stabilizes tissue-specific macrophage identity (80). Importantly, these changes are coupled to metabolic adaptations, including shifts in glycolytic and lipid metabolic pathways, which further support long-term persistence within chronically inflamed tissues.

Fate-mapping and reporter mouse studies have demonstrated that CCR2-derived monocytes can give rise to long-lived macrophage populations, including microglia-like or tissue-resident macrophages, which persist well beyond the initial inflammatory insult (9, 65, 71, 81). By redefining these cells through their state transitions, we can better understand how transient inflammatory influxes are converted into persistent, and potentially pathogenic, tissue niches. Importantly, the extent and directionality of this transition are highly dependent on local tissue context, inflammatory duration, and environmental signals, suggesting that CCR2-CX3CR1 dynamics are both plastic and reversible under certain conditions (65, 82). Emerging evidence suggests that tissue-adapted CX3CR1^+^ macrophage states are not irreversibly fixed, but may retain a degree of functional plasticity depending on the inflammatory microenvironment (65, 83). Resolution of inflammatory stimuli, restoration of tissue homeostasis, and removal of persistent microbial or cytokine-driven signaling may partially reverse macrophage activation states and reduce CX3CR1-associated inflammatory persistence (58, 84). Conversely, chronic inflammatory niches characterized by sustained cytokine production, hypoxia, stromal remodeling, and microbial dysbiosis may stabilize pathogenic CX3CR1^+^ macrophage populations through epigenetic and metabolic adaptation (85, 86). These observations suggest that reversibility of the CCR2-to-CX3CR1 transition is likely context-dependent and may be more achievable during early or transient inflammatory stages than in chronically established inflammatory tissues.

Together, these findings support our unifying recruitment-to-residency model, in which CCR2 and CX3CR1 define a recruitment-to-residency axis that governs myeloid cell fate and function. Within this unique framework, CCR2^+^ monocytes represent the entry state driven by systemic inflammatory signals, whereas CX3CR1^+^ cells reflect the tissue-adapted endpoint shaped by local microenvironmental cues. Understanding the breakdown of this coordinated axis is essential for deciphering the chronicity of diseases such as periodontitis.

## 4 Ligand biology and tissue microenvironment

Chemokine receptor signaling is fundamentally shaped by the spatial and temporal distribution of their ligands within tissue microenvironments (87). Beyond serving as simple chemoattractants, we define chemokine ligands as microenvironmental organizers that encode contextual information, integrating systemic inflammatory signals with local tissue-specific cues (88, 89). This integration is what ultimately directs immune cell positioning, retention, and functional adaptation.

In the context of the CCR2-CX3CR1 axis, the distinct biochemical properties and expression patterns of their respective ligands, CX3CL1 and CCL2, critically define the balance between immune cell recruitment and tissue residency (22, 46, 90). Rather than acting in isolation, these opposing yet coordinated ligand systems establish a spatial and functional axis, a ligand-driven landscape, that provides the essential mechanistic foundation for the CCR2-to-CX3CR1 state transition (23, 50). By linking early inflammatory mobilization driven by soluble CCL2 to long-term tissue adaptation and persistence mediated by membrane-bound and soluble CX3CL1, this microenvironmental regulation ensures that myeloid cells evolve in lockstep with the needs of the tissue, a process that becomes dysregulated in chronic inflammatory states (19, 65).

### 4.1 CX3CL1: a unique chemokine governing adhesion and retention

CX3CL1 (fractalkine) is a unique chemokine that exists in both membrane-bound and soluble forms, enabling it to function as both an adhesion molecule and a chemoattractant (22, 90). This dual functionality distinguishes CX3CL1 from traditional soluble chemokines and underlies its central role in mediating the residency phase of our proposed recruitment-to-residency axis (91, 92). Membrane-bound CX3CL1 is constitutively expressed by non-hematopoietic cells, including endothelial cells, epithelial cells, neurons, and stromal cells, where it facilitates firm adhesion of CX3CR1-expressing cells under shear flow conditions (93). By acting as a molecular anchor, membrane-bound CX3CL1 provides the physical scaffold necessary for infiltrating cells to stabilize their position and initiate functional tissue adaptation (25, 93).

Proteolytic cleavage of CX3CL1 by metalloproteinases such as ADAM10 and ADAM17 generates a soluble form that acts as a chemoattractant, further enhancing the recruitment of CX3CR1^+^ cells to sites of inflammation (94, 95). In the central nervous system, neuron-derived CX3CL1 regulates microglial activation and maintains neuroimmune homeostasis (96). In peripheral tissues, epithelial- and endothelial-derived CX3CL1 establishes localized niches that support the retention, survival, and functional specialization of CX3CR1^+^ macrophages (97–99). These CX3CL1-rich niches serve as the functional ‘destinations’ where the myeloid continuum reaches its endpoint, transitioning from an inflammatory influx to a settled, tissue-adapted state (**Figure 1**).

Importantly, CX3CL1 expression is dynamically regulated by inflammatory stimuli, including TNF, IL-1β, and oxidative stress, linking tissue damage to the stabilization of CX3CR1^+^ cell populations (91, 100). This inflammatory induction of CX3CL1 is a critical determinant of whether an immune response successfully resolves or transitions into chronic persistence (29, 101). Through these mechanisms, CX3CL1-CX3CR1 signaling acts as a critical determinant of tissue residency, enabling long-term immune surveillance, but also contributing to the persistence of pathogenic macrophages in chronic inflammatory diseases, a phenomenon that becomes a central driver of tissue destruction in the periodontal microenvironment.

### 4.2 CCL2 and CCR2 ligand redundancy: fuel for the initiation of the recruitment-to-residency axis

In contrast to CX3CL1, CCR2 ligands including CCL2, and related inflammatory chemokines, primarily function as soluble chemokines that establish the rapid chemotactic gradients required to ignite the myeloid continuum (46, 49). Among these, CCL2 is the most extensively studied and serves as a key regulator of inflammatory monocyte trafficking (45, 50). CCL2 is rapidly induced in response to tissue injury, infection, and inflammatory cytokines, and is produced by a wide range of cell types, including endothelial cells, fibroblasts, epithelial cells, and infiltrating leukocytes (10, 52, 60). Additional CCR2 ligands such as CCL7, and murine CCL12 in experimental models, may contribute to partial functional redundancy within the CCR2 signaling axis; however, CCL12 does not have a direct human ortholog.

CCR2 signaling is essential for the mobilization of the entry state population from the bone marrow, as demonstrated by studies showing impaired monocyte mobilization in CCR2-deficient mice (45, 102). Upon entering the circulation, CCR2^+^ monocytes follow chemokine gradients toward inflamed tissues, where high local concentrations of CCL2 and related ligands facilitate their recruitment and accumulation (49, 50). Notably, we highlight the functional redundancy within the CCR2 axis as a robust failsafe mechanism that ensures sustained monocyte recruitment even when individual chemokines are limited, thereby providing a constant influx of precursors for subsequent tissue adaptation (46).

Within inflamed tissues, persistent production of CCR2 ligands promotes continuous influx of inflammatory monocytes, thereby amplifying immune responses and contributing to chronic inflammation (50, 52). A unique feature of this initiation phase is that the subsequent downregulation of CCR2 expression following tissue infiltration represents a critical functional checkpoint (45, 50). This checkpoint enables monocytes to transition toward tissue-adapted states, often accompanied by increased CX3CR1 expression (9, 23). Thus, CCR2 ligand dynamics not only drive immune cell recruitment, but also the functional driver that indirectly shapes the entire differentiation trajectory of myeloid cells (**Figure 2**).

Taken together, the opposing yet coordinated ligand systems of CX3CL1 and CCL2 establish a spatial and functional axis, a coordinated chemokine signaling landscape, that governs the balance between immune cell recruitment and tissue retention. This ligand-driven microenvironmental regulation provides the essential mechanistic foundation for the CCR2-to-CX3CR1 transition, effectively linking early systemic inflammatory mobilization to long-term tissue adaptation and the eventual establishment of persistent pathogenic niches in diseases like periodontitis.

## 5 CX3CR1 in inflammatory diseases

The functional impact of CX3CR1 signaling follows a complex double-edged paradigm, where its role shifts from a homeostatic sentinel to a driver of chronicity depending on the inflammatory context. Within our proposed state-based framework, this duality is explained by the receptor’s role in governing the residency phase of the myeloid continuum, a process that is essential for health but can be pathologically hijacked in disease.

### 5.1 Protective roles in tissue homeostasis

CX3CR1-expressing macrophages play essential roles in maintaining tissue homeostasis across multiple organ systems (24, 32). Under steady-state conditions, CX3CR1 signaling supports immune surveillance, cellular longevity, and the niche-specific functional specialization that characterizes the successful endpoint of the recruitment-to-residency axis (25). In barrier tissues such as the gut and liver, CX3CR1^+^ macrophages restrict microbial dissemination, promote mucosal immune tolerance, and coordinate adaptive immune responses, including IgA production (33–35). In the central nervous system, CX3CR1-dependent signaling between neurons and microglia, the prototypical example of embryonic-derived tissue residency, regulates synaptic pruning and neuronal survival, thereby preserving neural circuit integrity (27, 103).

Beyond microbial control, CX3CR1^+^ macrophages contribute to tissue repair and homeostatic turnover by clearing apoptotic cells and regulating inflammatory resolution (23, 104). These functions highlight CX3CR1 not merely as a passive marker, but as a critical determinant of the tissue-adapted immune states (25, 29). By ensuring that recruited cells successfully transition into stable, homeostatic residents, CX3CR1 signaling serves to sustain physiological balance and prevent the progression of acute insults into excessive, uncontrolled inflammation (9, 65). Within our model, this homeostatic state represents the ‘ideal resolution’ of the myeloid trajectory (**Figure 1**).

### 5.2 Pathogenic roles in chronic inflammation

Despite its protective roles, CX3CR1 signaling can contribute to disease pathogenesis under conditions of chronic inflammation (105). Within our framework, this represents a maladaptive residency, where the transition intended for tissue repair instead results in the pathological stabilization of inflammatory cells (83, 106). Persistent CX3CL1 expression within inflamed tissues promotes the retention and survival of CX3CR1^+^ macrophages, leading to the accumulation of long-lived, potentially pathogenic cell populations that resist apoptosis and resolution signals (67, 107, 108).

In neurological disorders, dysregulated CX3CR1 signaling, a breakdown of its usually protective role in microglia, has been associated with aberrant microglial activation and neurodegeneration (29, 109). Similarly, in cardiovascular and metabolic diseases, CX3CR1^+^ macrophages contribute to plaque formation, tissue remodeling, and chronic inflammatory responses (105, 110). In these contexts, the very mechanisms that ensure long-term tissue residency become the drivers of disease chronicity.

Importantly, the pathogenic role of CX3CR1 is often context-dependent and linked to prolonged exposure to inflammatory cues. We argue that in such settings, the myeloid continuum does not reach a homeostatic endpoint; instead, CX3CR1^+^ macrophages shift from regulatory functions toward sustaining a self-perpetuating inflammatory niche. In such settings, CX3CR1^+^ macrophages may shift from regulatory or homeostatic functions toward sustaining low-grade inflammation, thereby reinforcing disease chronicity (69, 76, 105). These findings underscore a dual role for CX3CR1 in balancing tissue protection and pathological persistence, a phenomenon that we will further define as a central driver of the non-resolving nature of periodontitis.

## 6 Disease-dependent roles of CCR2: the double-edged sword of recruitment

The functional impact of CCR2 signaling is characterized by a temporal dichotomy, where its essential role in host defense can transition into a driver of pathological damage if not properly resolved (45, 50). Within our recruitment-to-residency axis, CCR2 serves as the ignition switch that launches the myeloid continuum, a process vital for acute survival but detrimental when chronically sustained.

### 6.1 Acute inflammation and host defense: the essential entry state

CCR2-mediated monocyte recruitment is a fundamental component of acute inflammatory responses and host defense (45). In response to infection or tissue injury, rapid induction of CCR2 ligands such as CCL2 drives the mobilization of inflammatory monocytes from the bone marrow and their recruitment to affected tissues (46, 111, 112). In our model, these CCR2^+^ monocytes represent the foundational entry state, specialized for rapid deployment to sites of stress. Once recruited, these cells differentiate into effector macrophages or dendritic cells that produce pro-inflammatory cytokines, reactive oxygen species, and antimicrobial mediators necessary for pathogen clearance and tissue repair (64).

In many experimental models, genetic deletion or pharmacological inhibition of CCR2 results in impaired monocyte recruitment and increased susceptibility to infection, highlighting its essential role in early immune responses (45, 46, 50, 113). These findings underscore that the CCR2 axis is not inherently pathogenic; rather, it is the indispensable first phase of the recruitment-to-residency axis that links systemic immune readiness to local tissue protection. Thus, CCR2 represents the kinetic engine of the initial inflammatory phase, providing the necessary precursor pool for subsequent tissue adaptation and resolution.

### 6.2 Chronic inflammatory diseases: dysregulation of the entry state

While CCR2-driven recruitment is beneficial during acute inflammation, sustained activation of this pathway contributes to chronic inflammatory pathology (50). Within our proposed framework, this chronicity represents a failure to resolve the initial entry state influx, transforming it into a continuous driver of tissue destruction. Persistent production of CCR2 ligands leads to continuous influx of inflammatory monocytes, resulting in the accumulation of pro-inflammatory macrophages within tissues (46). This process has been implicated in a wide range of diseases, including atherosclerosis (105), fibrosis (114), metabolic disorders (115), and mucosal inflammation (104).

In chronic settings, excessive CCR2-dependent recruitment amplifies tissue damage, disrupts resolution pathways, and sustains inflammatory feedback loops. Notably, from the perspective of the myeloid continuum, the prolonged presence of CCR2-derived monocytes also provides a perpetual source of precursor cells that differentiate into tissue-adapted macrophages, including CX3CR1^+^ populations (9, 19). This highlights that the recruitment-to-residency axis can be pathologically co-opted; instead of promoting resolution, it facilitates the establishment of persistent inflammatory niches, where recruited cells are stabilized in a chronically active state.

Together, these findings suggest that CCR2 and CX3CR1 function in a coordinated and sequential manner. Rather than seeing them as independent events, we define CCR2-driven recruitment and CX3CR1-mediated retention as functionally linked stages of a single trajectory. In this sequential model, the magnitude of the CCR2-driven influx establishes the inflammatory baseline, while the efficiency of the CX3CR1-mediated transition dictates the long-term tissue outcomes. As we will explore in the context of periodontitis, the breakdown of this coordinated axis is what ultimately prevents immune resolution and drives bone loss (**Table 1**).

**Table 1:** Integrated profile of the CCR2-CX3CR1 recruitment-to-residency axis and context-dependent outcomes.

Category	Entry state (CCR2^+^)	Residency state (CX3CR1^+^)
I. Biological Profile	Systemic Mobilization	Tissue Adaptation/Persistence
Major Markers	Ly6C^+^ (Mouse), CD14^+^ (Human)	CX3CR1^+^, CD16^+^ (Human)
Primary Ligands	CCL2 (MCP-1), CCL7	CX3CL1 (Fractalkine)
Signaling Pathways	PI3K-Akt, MAPK/ERK, NF-κB	PI3K-Akt, Src-family, Anti-apoptotic
Primary Function	Bone marrow egress and recruitment	Tissue retention, surveillance and homeostasis
II. Disease Context	CCR2-associated Role	CX3CR1-associated Role
Acute Infection	Rapid monocyte recruitment (Protective)	Microenvironmental surveillance (Protective)
Tissue Repair	Debris clearance/Inflammatory initiation (Protective)	Regenerative macrophage support (Protective)
Cardiovascular Disease	Monocyte recruitment and plaque inflammation	Foam cell persistence (Pathogenic)
Neuroinflammation	Systemic inflammatory influx	Microglial activation and persistence (Mixed)
Periodontitis	Potential osteoclast precursor recruitment	Persistent osteoclastogenic inflammatory niche
Overall Outcome	Acute inflammatory amplification	Persistent chronic inflammation (if dysregulated)
Therapeutic Strategy	Short-term modulation of inflammatory recruitment	Targeting macrophage persistence and chronic inflammatory niches

This table summarizes the transition of myeloid cells from an inflammatory entry state (CCR2^+^) to a tissue-adapted residency state (CX3CR1^+^), detailing key markers, and signaling pathways. It contrasts the protective roles of this axis in acute homeostasis with its pathogenic contribution to chronic diseases. Specifically, it highlights how dysregulated monocyte recruitment and macrophage persistence in periodontitis establish a non-resolving inflammatory niche that fuels osteoclastogenic bone destruction.

## 7 The CX3CR1-CCR2 axis in periodontitis: a prototypical model of non-resolving inflammation

Periodontitis is a chronic inflammatory disease driven by dysbiotic microbial communities and a dysregulated host immune response, ultimately leading to destruction of periodontal tissues and alveolar bone (116, 117). Within this unique framework of this review, periodontitis serves as a primary showcase for our recruitment-to-residency axis. The coordinated dynamics of CCR2-dependent monocyte recruitment and CX3CR1-mediated macrophage retention provide a mechanistic framework for understanding how transient inflammatory responses evolve into chronic, non-resolving pathology. By examining this axis, we can decipher how the breakdown of myeloid state transitions directly translates into clinical tissue destruction.

### 7.1 Periodontitis as a chronic immune-mediated disease: the failure of the resolution trajectory

Although microbial biofilms initiate periodontal inflammation, disease progression is largely determined by host immune responses, rather than direct bacterial damage (117, 118). Dysbiosis promotes sustained activation of innate and adaptive immune pathways, resulting in excessive production of inflammatory mediators such as TNF, IL-1β, and IL-6, which drive tissue destruction and bone resorption (119, 120).

From a state-based perspective, this chronic inflammatory microenvironment acts as a pathological driver that prevents the myeloid continuum from reaching a homeostatic endpoint (106). In addition, disruption of the gingival epithelial barrier facilitates microbial invasion and amplifies inflammatory signaling, creating a self-perpetuating cycle of immune activation and tissue damage (117, 121, 122). This chronic inflammatory microenvironment is characterized by persistent and dysregulated production of entry-state chemokines production, most notably the CCR2 ligand CCL2, which promotes a continuous and unchecked influx of inflammatory monocytes into the gingival niche (123).

Importantly, the CCR2-CX3CR1 axis should not be interpreted as an isolated upstream initiator independent of the microbial environment. Dysbiotic dental plaque biofilms provide the primary inflammatory trigger that induces persistent production of CCR2 ligands such as CCL2 within periodontal tissues. During reversible gingivitis, transient plaque accumulation promotes limited CCR2-dependent monocyte recruitment that may resolve following biofilm removal and restoration of epithelial barrier integrity. However, in chronic periodontitis, prolonged microbial stimulation sustains the recruitment-to-residency axis, resulting in the stabilization of pathogenic CX3CR1^+^ macrophage populations and the establishment of self-perpetuating inflammatory niches. Thus, dysregulation of the CCR2-CX3CR1 axis likely evolves from an initial downstream consequence of microbial dysbiosis into an autonomous driver of chronic inflammation, impaired immune resolution, and osteoclastogenic bone destruction.

### 7.2 CCR2-driven recruitment and CX3CR1-mediated retention: the synergy of chronic destruction

Within the periodontal microenvironment, the hyper-induction of CCR2 ligands acts as a persistent source, fueling the constant influx of inflammatory monocytes into gingival tissues. Elevated expression of CCR2 ligands in periodontal lesions drives the continuous recruitment of inflammatory monocytes into gingival tissues (46, 111). Dysbiotic dental plaque biofilms likely shape the CCR2-CX3CR1 axis through sustained activation of innate immune signaling pathways (117, 122). Periodontal pathogens and their associated PAMPs, including lipopolysaccharide, fimbriae, lipoproteins, and bacterial nucleic acids, activate gingival epithelial cells, fibroblasts, and resident immune cells through pattern-recognition receptors such as TLR2 and TLR4 (124, 125). This stimulation induces NF-κB-dependent production of inflammatory cytokines and chemokines, including TNF, IL-1β, IL-6, and CCL2 (46, 52, 120), thereby promoting persistent CCR2-dependent monocyte recruitment into periodontal tissues. Under conditions of chronic dysbiosis, prolonged inflammatory signaling and tissue damage further enhance CX3CL1 expression within the gingival microenvironment, supporting the survival, retention, and pathogenic adaptation of CX3CR1^+^ macrophages (67, 126). Thus, the plaque biofilm does not merely initiate inflammation, but actively sustains the recruitment-to-residency axis that underlies chronic periodontal destruction. These CCR2^+^ monocytes differentiate into macrophages and osteoclast precursors, thereby contributing to both inflammatory amplification and alveolar bone resorption (119, 127). A unique and critical feature of this entry state in periodontitis is that these CCR2^+^ monocytes do not merely amplify inflammation. They may serve as the primary precursors for both pathogenic macrophages and osteoclasts, directly linking myeloid recruitment to alveolar bone resorption.

Following tissue infiltration, recruited monocytes undergo phenotypic and functional reprogramming in response to local cytokines, microbial products, and damage-associated signals (9, 65). This *in situ* reprogramming constitutes the mechanistic heart of the recruitment-to-residency axis, where infiltrating cells progressively exchange their migratory readiness (CCR2) for a program of local persistence (CX3CR1) (21, 46, 128).

In periodontal disease, this transition becomes pathologically skewed toward persistent inflammatory macrophage states. Increased CX3CL1/CX3CR1 signaling promotes prolonged macrophage survival, bacterial persistence and sustained osteoclastogenic inflammation within periodontal tissues (129–131). Importantly, CX3CR1^+^ macrophages should not be regarded as exclusively pathogenic populations. Under homeostatic or early inflammatory conditions, CX3CR1-expressing macrophages contribute to microbial surveillance, bacterial restriction, and maintenance of tissue barrier integrity (33, 35, 38). Previous studies have demonstrated that CX3CR1-dependent myeloid cells can limit microbial dissemination and coordinate local innate immune responses in mucosal tissues (33, 35, 39). However, within chronically dysbiotic periodontal environments, persistent microbial stimulation may reprogram these cells toward maladaptive inflammatory states characterized by prolonged survival, incomplete bacterial clearance, and sustained production of osteoclastogenic mediators (117, 129, 131). Consequently, although CX3CR1^+^ macrophages may partially restrict plaque-associated bacteria, their long-term retention within inflamed periodontal tissues can paradoxically promote chronic inflammation and alveolar bone destruction.

Clinical evidence reinforces this model, identifying CX3CL1 and CX3CR1 as potent biomarkers that track with the severity of periodontitis and its systemic comorbidities, such as rheumatoid arthritis (126, 132). These observations further provide strong validation for our proposal that these receptors are indicators of a dynamic, disease-driving state.

Mechanistically, the danger in periodontitis lies in the CX3CL1-mediated stabilization of these cells. CX3CR1 signaling promotes the survival and long-term persistence of macrophages within inflamed tissues (9, 10, 67). We define this phenomenon as the formation of a self-perpetuating inflammatory niche, which is a localized microenvironment where sustained CX3CL1 expression traps pathogenic macrophages, preventing immune resolution and ensuring that the inflammatory cycle remains unbroken.

Taken together, these findings support our sequential model, in which CCR2-driven recruitment provides the fuel, a continuous influx of inflammatory monocytes, while CX3CR1-mediated retention provides stability (persistence). In our unique framework, this recruitment-to-residency axis represents the critical checkpoint that its failure to reach a homeostatic conclusion ultimately dictates the shift from reversible gingivitis to chronic, destructive periodontitis. Clinical improvement following periodontal therapy is frequently associated with reduced inflammatory chemokine production and partial restoration of immune homeostasis, suggesting that the CCR2-CX3CR1 axis remains dynamically regulated and potentially reversible during early disease stages (**Figure 2**).

### 7.3 System integration and conceptual parallels: the osteoimmune nexus

**Figure 3:**
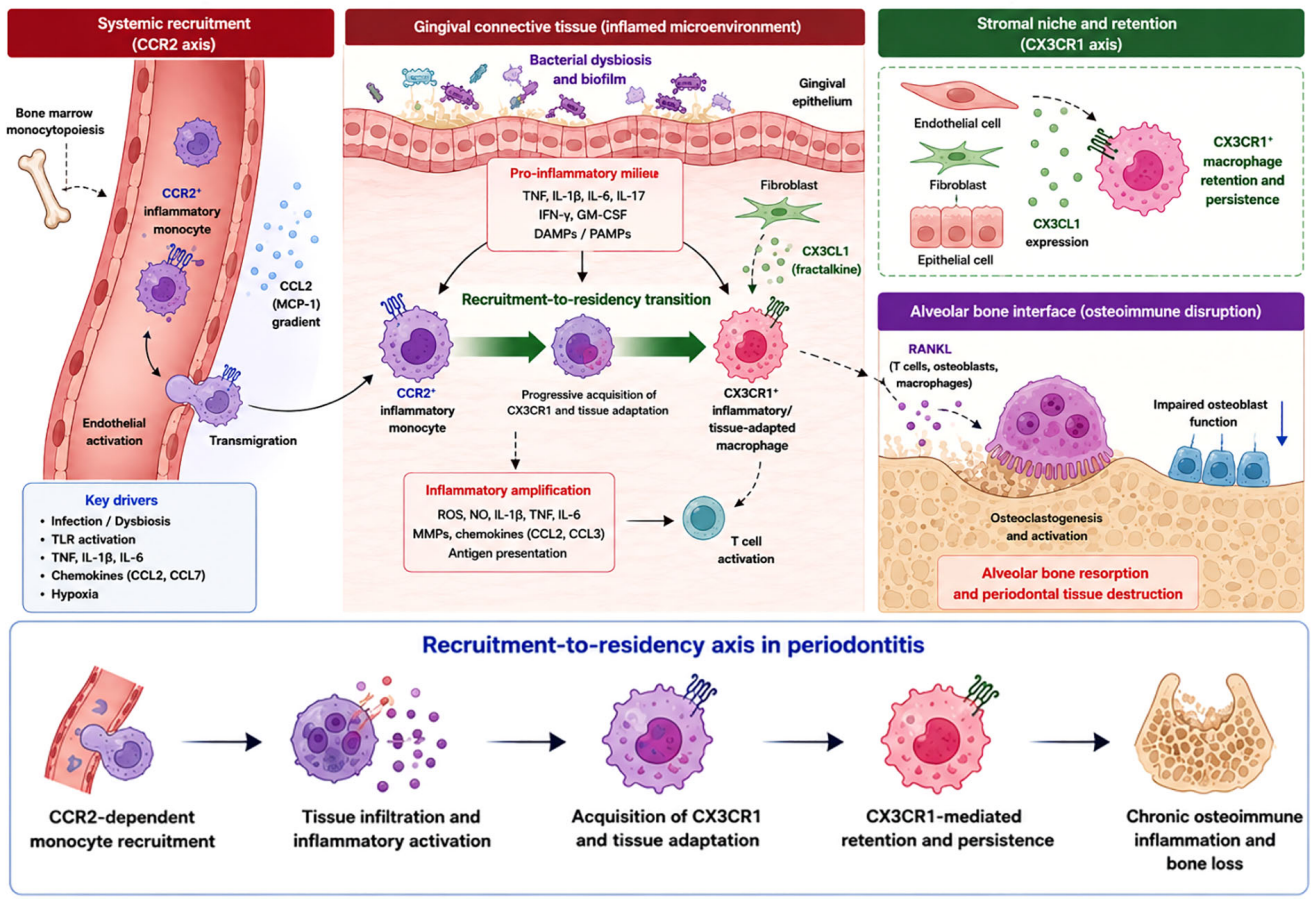
Spatial organization of The CCR2-CX3CR1 recruitment-to-residency axis in periodontitis. Schematic illustration of the dynamic transition from CCR2^+^ inflammatory monocyte recruitment to CX3CR1^+^ tissue-adapted macrophage persistence within periodontal tissues. Dysbiotic biofilms and inflammatory mediators promote CCR2-dependent monocyte infiltration into gingival tissues, followed by progressive acquisition of CX3CR1-associated tissue residency programs. Persistent CX3CR1^+^ macrophage accumulation contributes to inflammatory amplification, osteoclastogenic signaling, impaired osteoblast activity, and alveolar bone destruction.

In periodontal tissues, CCR2-CX3CR1 dynamics represent more than just a trafficking pathway; they intersect with osteoclast-mediated bone resorption, neutrophil recruitment, and adaptive immune responses (133–135). This intersection highlights the seamless integration of myeloid recruitment, long-term retention, and tissue remodeling (**Figure 3**).

A unique strength of our recruitment-to-residency framework is its ability to bridge the gap between immune cell state and skeletal outcomes. In periodontitis, the continuous CCR2-driven influx provides a potential source of osteoclast precursor populations (136, 137), while the subsequent CX3CR1-mediated stabilization of these cells within the gingival niche may contribute to the establishment of a persistent osteoclastogenic environment (138). Persistent recruitment of CCR2^+^ inflammatory monocytes also increases the local accumulation of cytokine-producing macrophages that secrete TNF, IL-1β, and IL-6, which enhance RANKL-mediated osteoclast differentiation and activation (119, 139–141). However, the precise cellular origin of osteoclasts within periodontal inflammatory lesions remains incompletely resolved and continues to be debated. While multiple studies support a significant contribution of CCR2^+^ inflammatory monocytes as osteoclast precursors during periodontal inflammation (46, 142), other evidence suggests that CX3CR1^+^ tissue-adapted macrophages may also directly contribute to osteoclast differentiation under chronic inflammatory conditions (143). These discrepancies may reflect differences in tissue context, inflammatory stage, experimental models, and lineage-tracing methodologies (19, 72). Therefore, although our recruitment-to-residency axis proposes that dysregulated myeloid state transitions contribute to osteoclastogenic inflammation, the relative contribution of CCR2^+^ monocytes versus CX3CR1^+^ macrophage populations to osteoclast formation in periodontitis remains to be definitively established. Future fate-mapping and lineage-tracing studies will be essential to clarify these cellular relationships *in vivo* (9, 65). As these infiltrating monocytes transition toward CX3CR1^+^ tissue-adapted macrophage states, prolonged macrophage survival and retention may further stabilize osteoclastogenic inflammatory niches within periodontal tissues (33, 67, 68). In addition to promoting osteoclast activity, chronic inflammatory signaling associated with persistent CX3CR1^+^ macrophage accumulation may impair osteoblast differentiation and bone-forming capacity, thereby disrupting normal bone remodeling and limiting regenerative repair (144–146). Persistent activation of CCR2-CX3CR1-associated signaling pathways may therefore sustain inflammatory amplification loops that couple immune persistence to osteoclastogenic tissue destruction (**Figure 3**).

Importantly, several aspects of this proposed recruitment-to-residency axis remain conceptual and require further experimental validation, particularly in the context of periodontitis. While CCR2-dependent monocyte recruitment is well established, the precise mechanisms by which CX3CR1-associated macrophage persistence directly influence osteoclast differentiation, osteoblast suppression, and irreversible periodontal tissue destruction remain incompletely defined. Therefore, the models proposed here should be interpreted as integrative inferences based on currently available immunological and osteoimmune evidence rather than fully established pathological pathways.

Conceptually, these dynamics parallel neuroinflammatory settings, where CCR2-dependent monocyte influx and CX3CR1^+^ microglia-like populations modulate tissue inflammation and repair (147–149). By drawing this parallel, we elevate periodontitis to a prototypical status alongside neurodegeneration, where the breakdown of the recruitment-to-residency axis may represent a shared mechanistic framework contributing to chronic tissue destruction (117). This cross-disciplinary integration underscores that targeting the transition points between these states, rather than individual cell types, offers a superior strategy for achieving immune resolution across both oral and systemic compartments (83, 150).

### 7.4 Therapeutic implications: precision modulation of the recruitment-to-residency axis

Targeting the CCR2-CX3CR1 axis represents a promising strategy for modulating chronic inflammatory diseases, including periodontitis (116). Pharmacological inhibition of CCR2 has been shown to reduce monocyte recruitment and attenuate inflammatory responses in preclinical models (113, 151, 152). However, this review emphasizes that given the essential role of CCR2 in host defense, complete blockade may impair protective immune responses and increase susceptibility to infection (50, 64). Consequently, we propose that CCR2-targeted therapies should be strategically timed to the early mobilization phase or acute exacerbations of the myeloid continuum.

Importantly, accumulating evidence from chronic inflammatory disease studies suggests that targeting upstream chemokine recruitment pathways alone may provide only limited therapeutic benefit (153, 154). Clinical and preclinical studies using single chemokine receptor antagonists, including CCR2 inhibitors, have frequently demonstrated incomplete or transient efficacy, particularly in chronic inflammatory settings characterized by established tissue-resident immune populations (155). These limitations likely reflect the inability of recruitment-targeted therapies alone to eliminate persistent tissue-adapted macrophage niches that continue to sustain local inflammatory signaling even after inflammatory cell influx is reduced.

Conversely, targeting the CX3CL1-CX3CR1 axis may preferentially affect the persistence and retention of tissue-adapted macrophage populations that sustain chronic inflammation (67). Experimental studies suggest that disruption of CX3CR1 signaling reduces macrophage survival and tissue accumulation, thereby limiting long-term inflammatory responses (11). Several experimental approaches have been explored to disrupt this axis, including neutralizing antibodies against CX3CL1 or CX3CR1, small-molecule CX3CR1 antagonists, genetic deletion or silencing strategies, and modulation of downstream inflammatory signaling pathways associated with macrophage survival and tissue retention (68, 69, 156–159). These interventions have been shown to reduce inflammatory cell accumulation, attenuate chronic tissue inflammation, and limit pathological tissue remodeling in multiple preclinical inflammatory disease models (67, 105). However, because CX3CR1 signaling also contributes to immune surveillance and tissue homeostasis, excessive or prolonged blockade may impair protective immune functions and tissue repair (33, 38). In the context of periodontitis, this approach may be particularly effective in dismantling established self-perpetuating inflammatory niches characterized by CX3CR1^+^ macrophage accumulation (117).

Importantly, emerging clinical evidence indicating that CX3CL1 and CX3CR1 function as biomarkers of disease severity further highlights their potential as precision therapeutic targets (126, 132, 160, 161). These findings suggest that patient stratification based on CX3CL1-CX3CR1 activity may enable more tailored immunomodulatory strategies that specifically target the persistent inflammatory myeloid states in individual patients.

Taken together, these observations indicate that CCR2 and CX3CR1 represent temporally distinct but mechanistically linked therapeutic targets. Rather than relying on indiscriminate inhibition of a single chemokine pathway, effective intervention may require coordinated and stage-specific modulation of both inflammatory recruitment and tissue-resident persistence within the recruitment-to-residency axis. This conceptual framework may help explain the limited efficacy observed with single chemokine receptor blockade in chronic inflammatory diseases while highlighting the potential advantages of simultaneously targeting both recruitment and residency programs to restore immune homeostasis (153). Combination strategies targeting both recruitment and residency programs may provide synergistic therapeutic effects by simultaneously limiting inflammatory monocyte influx and disrupting persistent tissue-adapted macrophage niches (72, 154). However, excessive suppression of CCR2-dependent recruitment may impair antimicrobial host defense, while prolonged inhibition of CX3CR1-associated tissue residency could interfere with immune surveillance, tissue homeostasis, and regenerative repair (45, 71). These considerations highlight the importance of temporally controlled and context-dependent therapeutic modulation within the recruitment-to-residency axis.

## 8 Conclusion and future perspectives

The CCR2-CX3CR1 axis represents a fundamental organizing principle of myeloid cell biology that links inflammatory recruitment to tissue adaptation and long-term persistence (50, 162). Moving beyond traditional lineage-based classifications, this review redefines CCR2 and CX3CR1 as indicators of a dynamic and context-dependent myeloid states shaped by local microenvironmental cues (18). Within this framework, CCR2^+^ monocytes function as a systemic entry state, while CX3CR1^+^ macrophages represent the tissue-adapted endpoint specialized for long-term residency and immune surveillance (**Figure 1**).

This recruitment-to-residency axis provides a unifying conceptual model for understanding how acute inflammatory responses transition into chronic disease states. Across diverse pathological conditions, including neurological, cardiovascular, metabolic, and mucosal diseases, dysregulation of this axis contributes to the accumulation of long-lived, functionally specialized myeloid populations that sustain inflammation and impair resolution (50, 162) (**Table 1**). In the specific context of periodontitis, we highlight this axis as the primary mechanistic driver of the non-resolving inflammatory cycle, where continuous CCR2-driven monocyte influx and CX3CR1-mediated retention cooperate to establish a self-perpetuating inflammatory niche that directly fuels alveolar bone loss (**Figure 2**). Integration of this axis with osteoimmunology and neutrophil-driven inflammation further highlights the complex cellular networks that underlie tissue destruction and bone loss in chronic oral disease (141) (**Figure 3**).

Future studies integrating single-cell transcriptomics, spatial profiling, and lineage tracing will be essential for resolving the precise temporal and spatial dynamics of CCR2-CX3CR1 transitions across tissues. Single-cell RNA sequencing and spatial transcriptomic approaches may help resolve unresolved questions regarding the initiation and reversibility of CCR2-to-CX3CR1 state transitions. Furthermore, exploring how metabolic reprogramming, epigenetic remodeling, and microbiome-derived signals influence myeloid states will provide deeper insights into the plasticity of this axis. From a therapeutic perspective, future approaches should prioritize the restoration of balanced myeloid cell dynamics, state-turning, rather than the indiscriminate inhibition of individual receptors. Ultimately, redefining myeloid cell biology through the lens of dynamic myeloid state transitions may provides a conceptual foundation for the development of precision immunomodulatory therapies across oral and systemic inflammatory disorders.
